# Cognitive control, intentions, and problem solving in skill learning

**DOI:** 10.1007/s11229-022-03920-7

**Published:** 2022-11-02

**Authors:** Wayne Christensen, Kath Bicknell

**Affiliations:** 1 Philosophy, University of Barcelona, Barcelona, Spain; 2 School of Social Sciences and School of Psychological Sciences, Macquarie University, Sydney, Australia

**Keywords:** Skill learning, Problem solving, Cognitive control, Intentions, Action strategies

## Abstract

We investigate flexibility and problem solving in skilled action. We conducted a field study of mountain bike riding that required a learner rider to cope with major changes in technique and equipment. Our results indicate that relatively inexperienced individuals can be capable of fairly complex 'on-the-fly' problem solving which allows them to cope with new conditions. This problem solving is hard to explain for classical theories of skill because the adjustments are too large to be achieved by automatic mechanisms and too complex and rapid to be achieved by cognitive processes as they are usually understood. A recent theory, Mesh, can explain these results because it posits that skill-specific cognitive abilities develop during skill learning and that control typically involves an interplay between cognitive and automatic mechanisms. Here we develop Mesh further, providing a detailed explanation for these problem solving abilities. We argue that causal representation, metacognitive awareness and other forms of performance awareness combine in the formulation and control of action strategies. We also argue that the structure of control present in this case is inconsistent with Bratman's model of intentions, and that, in the face of high uncertainty and risk, intentions can be much more labile than Bratman recognises. In addition, we found limitations and flaws in problem solving which illuminate the representations involved. Finally, we highlight the crucial role of social and cultural learning in the development of complex skills.

## Introduction

What is learned during skill learning? What role does cognitive control—the form of control involved in flexible, goal-directed thought and action—play in the learning process? Classical skill theories, such as those of Fitts and Posner (1967) and Anderson (1982), treat cognitive control as responsible for discovering the structure of the actions that the skill requires, and for their initial implementation, but as being supplanted by more efficient automatic processes as learning progresses. Most work on skill focuses on the development of automaticity and the abilities and mechanisms that automatically-produced skills might involve. But humans show an exceptionally high degree of *flexibility* in skilled action, including forms of flexibility that rely on problem solving to construct solutions, as opposed to the deployment of pre-learned solutions. This kind of flexibility has not received much attention, even though it is arguably *the* critical ability underlying the richness and diversity of human skill.

The classical view sees skill as automating because it regards cognitive control as fundamentally unsuited to the demands of skilled action control. Cognitive control is thought to be slow, serial, and as having limited capacity, whereas automatic processes are fast, parallel, and have high capacity (Shiffrin & Schneider, 1977; Evans & Stanovich, 2013). Cognitive control uses highly generalised representations and problem solving methods which are an inefficient means for producing the specialised responses of skill (Anderson, 1982). In other words, cognitive control is specialised for reasoning, not action control, and it is a clumsy tool to use for action control. But it's questionable whether cognitive control is really fundamentally unsuited to skilled action control, as assumed by the classical view. Certainly, early cognitively-driven efforts to perform a skill are clumsy, and working memory is often overloaded by task demands. But significant degrees of fluency emerge long before strong automaticity could be in place. Skill research tends to focus on motor skill in particular, but if we consider expertise research more generally it is clear that experts can acquire domain-specific *cognitive* skills which can allow them to rapidly process large amounts of information (Ericsson & Kintsch, 1995) and engage in powerful, domain-specific forms of problem solving (Chi et al., 1982). There is no obvious reason why skills that involve a strong motor component might not also incorporate cognitive abilities involved in control and problem solving.

A recent skill theory called *Mesh* proposes just this (Christensen & Sutton, 2018; Christensen et al., 2016, 2019). It claims that almost all skills incorporate an important cognitive component, including those which are paradigmatically motoric like golf putting. Skilled performance is produced by meshed cognitive and automatic processes which are generated by the cooperative operation of many neural systems. Cognitive processes provide flexibility by shaping action to the context and by solving the problems that complex, variable environments and tasks present. This paper extends Mesh by investigating the nature of skilled problem solving more closely. We conducted a field study investigating adjustments to major task changes by a rider with several years mountain bike riding experience (and many years of road riding experience) but beginner-level mountain bike technique. This was one of the authors, Wayne. Kath, who is a highly experienced rider, provided instruction. Guidance from Kath prompted major changes in Wayne's riding technique. A change in bike during the field study also encouraged significant adjustments in Wayne’s riding.

Because Wayne was not a raw novice he possessed mountain-biking-specific problem solving abilities which allowed him to cope with these large changes more fluently than might be expected based on the classical view. His experiences navigating rocky descents and ascents, and challenging log roll-overs, help to illuminate the nature of the control involved in this kind of problem solving. These problems involve high uncertainty and significant risk, and control processes flexibly adjust action to manage this risk and uncertainty. The learner is learning the structure of the problem as they try out solutions, and the strategy is monitored, modified and sometimes abandoned during execution. Evaluation is complex, employing a rich set of criteria and flexible holistic assessment. To understand these features of control we need a more expansive concept of control and a more labile picture of intentions than the standard picture recognises (Bratman, 1987).

The problem solving that Wayne engaged in also helps to illuminate the nature of the representations involved. Recently there has been considerable interest in characterising the type of the representations that are required for the ability to perform actions (Pacherie, 2011). Most accounts have focused on representations of action *form*, with an emphasis on schemas (e.g. Mylopoulos & Pacherie, 2018; Fridland, 2021). In contrast, some accounts have focused on the representation of causal structure (Christensen et al., 2015; Goldenberg, 2013).

Here we endorse the view that causal representation plays an important role, and show how this kind of representation supports flexibility. We suggest that Wayne employed causal representation to identify the structure of the problems he faced and formulate solutions. Because he could understand the causal significance of some of the features of the altered technique and equipment he could rapidly formulate new strategies to cope with and exploit the changes. But Wayne's problem solving abilities showed strong limitations which are also revealing. Wayne failed to properly implement a key riding technique even though he understood the technique abstractly and thought he was implementing it. The problem was diagnosed by Kath, who corrected his implementation. His difficulty involved a failure to properly map between an abstract representation of the technique and the representations used in the control of execution. The latter have particular a type of content, in particular a systematic representation of the *state space of execution control*. They also have a characteristic perspective that we call *the perspective of execution control*. This perspective is related to, though distinct from, the type of perspective described by (Pavese, 2019) in her concept of practical modes of presentation.

In Sect. 2 we present the theoretical context for the study and describe its conception. In Sect. 3 we give an overview of the activities we conducted. Section 4 then analyses these experiences in close detail using the theoretical ideas developed in Sect. 2.

## Context and approach

### Metatheory

This paper centers on a field study which is analysed for its theoretical implications. In both philosophy and psychology this is an unusual method which requires some explanation and defense. In a separate paper, Christensen (in preparation) develops a general argument that cognitive ecology should be a central discipline in psychology, that ecological methods should be incorporated into philosophy just as experimental methods have been, and that the present lack of attention to cognitive ecology is a serious limitation on the development of deep theory in both psychology and philosophy of mind (see also Bicknell & Sutton, 2022). Here we focus on a more restricted argument which highlights some of the ways that the theoretical issues we are concerned with in this paper are sensitive to ecological data.

As noted, we are concerned with skill learning, and with the role of cognitive control, intentions, and action strategies in skill learning. These are evolved adaptive traits whose structure and function are shaped by a complex mixture of evolutionary adaptation and learning. It follows that understanding the nature of the adaptive functions and mechanisms depends critically on understanding the ecological problems they respond to. Consequently, we need to investigate these phenomena in the context of the ecological problems they are adapted to solve. This requires the use of ecological methods to investigate the nature of these problems. We currently lack a detailed understanding of these ecological problems.

Bratman's (1987) theory of intentions is an especially relevant case where theory is sensitive to the details of these ecological problems.^1^ Bratman’s account is widely accepted and is used as a framework by numerous contemporary researchers. Mylopoulos and Pacherie’s (2018) account of intentional action control is an example which we will compare with our own model. Bratman characterises intentions based on an analysis of human planning behavior. In developing his theory Bratman focuses primarily on "ordinary, humdrum cases in which future-directed intentions and partial plans lead without great difficulty from prior deliberation to later conduct" (p. 12). He sees these kinds of cases as contrasting with more complex cases that involve difficulties of self-control. He writes,

Such examples are quite fascinating. But I think *we get a distorted view of future-directed intention* if we take them as paradigmatic of intention. It is best, I think, to begin with ordinary, garden-variety cases in which, without major psychological resistance, future-directed intentions and partial plans support coordination in the lives of limited agents like us. It is *here that we need to look to get at the major regularities, roles, and norms in terms of which we can understand intention and its associated commitment*. (p. 12, emphasis added.)

In this passage Bratman shows an awareness of the importance of the ecological representativeness of types of cases. However, Bratman's ecological picture is itself flawed. Bratman appears to assume that ordinary humdrum cases “without great difficulty” are ecologically typical or predominant while cases with major psychological conflict are exceptional. He also assumes that these are the only significant variations to be considered. Bratman's examples are indeed, for the most part, mundane, such as going to the library to borrow a book, or deciding whether to have a milkshake for lunch. These cases involve low stakes, high information, low complexity, context stability, and low time pressure. Only one of his examples, a presidential TV debate, involves high stakes, high complexity and high time pressure. Bratman uses the complexity and time pressure of the case in his analysis but he doesn't systematically examine these features as such or consider the significance of variation in them for his theory. All these features vary greatly across naturally occurring human ecological contexts. And crucially, these variations have important theoretical implications. The basic structure of Bratman’s model of intention involves a phase of deliberation in which an intention is formed, followed by a phase in which evaluation of the intention has ceased and cognitive processes are devoted to implementation. Intentions serve as fixed anchor points which structure cognition and behavior after they have been adopted. But this model may be best suited to cases that involve low stakes and high information. In cases with significant uncertainty and high stakes we might expect evaluation of the intention to be ongoing during execution as information comes in. In some kinds of cases it is possible to abort an action part way through or change its fundamental nature. Accordingly, in these kinds of contexts intentions may be relatively labile, being evaluated, modified, and sometimes abandoned during execution as information about the action is acquired. Skill learning is an important context in which this kind of pattern is likely.

Lack of ecological data is also a problem for skill theory. Classical skill theories, such as those of Fitts and Posner (1967) and Dreyfus and Dreyfus (1986), propose that skill acquisition has a well-defined stage structure culminating in a final stage of full automaticity. Fitts and Posner's model has just three stages, while Dreyfus and Dreyfus's account has five. Yet skills are extremely diverse, ranging from the ability to play tic-tac-toe to being a concert pianist or astrophysicist. It is unlikely that a simple three or five stage model provides a satisfactory fit to all the skill acquisition pathways involved in developing such diverse expert abilities. In athletics, high jumping has a standardised, invariant task structure, and a single action strategy, the Fosbury Flop, has dominated since the late 1960s. In contrast, in competition bouldering route setters set highly diverse novel climbing problems at each competition. Climbers are allowed only four minutes to inspect the wall and formulate strategies. Climbers try out varied strategies, often employing strategies which suit their individual abilities and physical characteristics, such as explosive strength, limb and hand size, or flexibility and balance.^2^ Multiple strategies may succeed on a given problem, including strategies not anticipated by the route setters.^3^ At odds with classical skill theories, climbers and commentators often describe bouldering at elite levels as highly cognitive. This is less often said about high jump. To get a better understanding of skill we need a fuller picture of this diversity.

In this paper we take a small step towards filling out this picture. Our objective is to bring detailed theoretical analysis into a close engagement with detailed empirical ecological reporting and analysis. While our sample is a tiny slice of the big picture, close analysis reveals that it has features with wide-ranging theoretical significance.

### Theory

#### Ecological context

The ecological context we have focused on is that of socially-guided learning of a complex, fast-paced skill in a variable, physically demanding environment. Specifically, we have focused on the problems of coping with major changes in mountain biking technique and equipment in an individual at a relatively early stage of skill development, with some experience but beginner-level technique. The ability to acquire complex, fast-paced sensorimotor skills is highly developed in primates, with arboreal lifestyle being a primary ecological basis for this. Primates show high levels of behavioral flexibility, manifested in foraging strategies, communication, social behavior and tool use. Amongst primates, humans are exceptional in showing an extremely highly developed capacity for flexible skill acquisition. This plays a fundamental role in human lifestyles. Human skills tend to be highly social, often acquired through social learning and exercised in social contexts.

Cognitive control plays a central role in learning complex novel skills. It is accordingly likely that in human evolution there has been selection on the capacity for cognitive control for functions that contribute to complex skill learning. Some contributions of cognitive control are probably not specific adaptations for skill learning, but rather more general abilities that contribute to skill learning amongst other important adaptive human capacities. Conversely, skill learning is likely to shape the mechanisms of cognitive control generally, both via selection and activity-dependent plasticity. During skill learning, new capacities for cognitive control are acquired, so some of the capacities and mechanisms involved in cognitive control may be more apparent (see for example, Bicknell, 2021; Bicknell & Brümmer, 2022; Downey, 2022).

Our rationale for focusing on the ability to cope with major changes in technique and equipment is that this is a demanding context which should illuminate mechanisms for control of performance and skill learning.

#### Testing the classical procedural-cognitive contrast

We can make an initial framing in terms of the standard distinction between implicit and explicit processes. Broadly, the kind of *procedural* or *implicit* learning usually associated with skill acquisition occurs slowly and incrementally, and the abilities which result are relatively inflexible (Reber et al., 2019). These mechanisms should hence be unable to respond to large, rapid changes. In addition, the classical view of skill claims that cognitive processes are ill-suited to the control of skilled action. Cognitive control processes should therefore struggle to cope with major novel changes to the way a task is performed. In contrast, Mesh theory claims that cognitive control of action improves with skill learning and incorporates several features which allow relatively efficient control, including representation of the causal structure of action problems and metacognitive and other kinds of performance awareness (Christensen et al., 2015, 2019). It is consequently better placed to explain relatively fluent adjustments to major novel changes if, as we expected, these do occur.

#### Forms of flexibility

We also need a more fine-grained framing of the rationale for looking at large changes in technique and equipment. This is because numerous motor control mechanisms have been proposed which are capable of flexibility in various forms. In the situations we're concerned with it is plausible that multiple forms of flexibility play important roles.

One of the simplest forms of flexible control is feedback control. Here, feedback corrects deviation from a reference. The goal can be achieved from any point in a state space 'basin' defined by the abilities to detect deviations and produce control inputs which drive the system towards the goal state. *Trace theory* (Adams, 1971) and the *control law* model (Fajen, 2005; Gibson, 1979) are theories of skilled action production based on feedback. Another class of control system achieves greater flexibility by means of generalised sensorimotor mappings. Theories of this type include Schmidt's (1975) *schema theory*, the *internal models* approach (Daniel M. Wolpert & Kawato, 1998; Wolpert et al., 1995), *Optimal Feedback Control* theory (Todorov, 2004), and the coordinative structures of *dynamical systems theory* (e.g. Kelso & Zanone, 2002), which generate high order patterns in movement. The perception-motor mappings are generalised in the sense that they generalise from practiced to unpracticed contexts based on similarity.

*Calibration* is a form of flexibility in which the parameters of a control function are adjusted to suit the context. A different kind of flexibility is achieved by restricting regulation only to variables that affect goal-achievement (Todorov, 2004; Tseng et al., 2003). Restricting control only to variables that affect goal-achievement is resource-efficient and can have the effect of decoupling variables important for the goal from those which aren't, which buffers performance against variations in unimportant variables. Yet another kind of flexibility involves control of the way a strategy is executed. *Impedance control*, or the control of the stiffness of the motor system, is an example (Franklin et al., 2008). Thus, a given action type can be performed while maintaining varying degrees and forms of stiffness. Control of stiffness can have a variety of functional benefits. For instance, increasing stiffness can reduce the degrees of freedom present in a movement and hence simplify the movement problem, while reducing stiffness can reduce the negative consequences of impacts that arise as a result of errors.

A key form of flexibility, sometimes called *equivalence*, involves the ability to achieve a given goal using multiple qualitatively distinct movement patterns. Ranganathan et al. (2020) identify two kinds of mechanism capable of this kind of flexibility. The first type involve high order task-specific *coordination functions* which constrain the dynamics of the system in a way that allows multiple coordination modes, or *synergies*. The second consist of *explicit strategies* (Christensen & Bicknell, 2019; Christensen et al., 2015, 2019; Shepherd, 2017; Taylor & Ivry, 2012). Ranganathan et al. (2020) suggest that flexibility is likely to be based on synergies when the variations in movement pattern are relatively small and the task constraints can be learned over a long period of time. Explicit strategies are likely to be used when the variations in movement patterns are large and the task constraints change over short time scales. The situation we are examining has these features so it should evoke the use of strategies.

#### Problem solving

The key question that then arises with regard to action strategies is how they are formulated and controlled using cognitive processes. As we saw, according to the classical view (e.g. Anderson, 1982) cognitive control includes no specialisations for action control. An alternative view, adopted in Mesh, is that control of action is one of the primary functions performed by cognitive control, and it incorporates mechanisms acquired through evolution and learning that are specialised for this role. These mechanisms engage in problem solving processes which represent the structure of action problems and construct solution strategies. Cognitive control then governs the implementation of these strategies.

Problem solving is a relatively understudied issue in motor control research, which is surprising on ecological grounds given the high degree of diversity and flexibility of human motor abilities, and the importance of flexible motor abilities in human evolution. Bernstein's (1996) concept of *dexterity* is an exception to this neglect. Bernstein characterises dexterity as the ability to find solutions to novel motor problems, and he regards it as central to human motor ability. Dexterity in this sense is likely to have fairly deep evolutionary roots, being important for locomotion in arboreal primates, for example. A recent study of squirrels illuminates some of the kinds of motor problem solving that an arboreal lifestyle involves, including adjusting to the flexibility of branches, distances, and the three-dimensional configuration of space and surfaces.^4^ Human dexterity shows greatly enhanced range and depth, in the sense that humans are able to solve a much wider variety of motor problems and much more complex problems (Gibson 1979).

#### Causal representation and problem solving

Mesh treats the capacity for flexible problem solving as central to human skill and proposes that it incorporates three key ingredients. Firstly, there is the ability to flexibly represent problems as causally structured wholes by means of *causal models*. These represent problems as structured wholes incorporating constituents and relations. At least some constituents must be represented as able to vary in state, requiring a distinction between variables and the values that variables can take. We will refer to some variables as *parameters*, where by this we mean key features of a type, such as an action type. The representation of causal relations requires that parameters are represented as related by *production* relations, such that, in the simplest case, a change in the of state of a particular parameter produces a change in the state of a second parameter.

Most accounts of action representation have focused on the representation of action *form*, such as motor patterns, schemas and automated procedures (Anderson, 1982; Buxbaum, 2001; Fridland, 2021; Pacherie & Mylopoulos, 2020; Schmidt, 1975; Wolpert et al., 1995). These theories propose that, when provided with a goal in a particular context, the motor system predicts what action structure will achieve the goal and then produces that structure. Crucially, there is no representation of the causal relation between the action structure and the goal, or between components of the action structure. In contrast, causal theories claim that action control incorporates explicit representation of causal relations. For instance, the individual might use awareness of the weight of an object in order to estimate how much force to use in picking it up, or awareness of the mechanical properties of a knife blade to control its manipulation when using it as a prying lever. Thus, Goldenberg (2013) proposes that action control employs a mechanical problem-solving system that represents objects and the body in terms of parts and properties relevant to action problems. Pavese (2021) argues that these representations of causal principles are practical concepts, or concepts used for intentional control of action. Somewhat similarly to Goldenberg, we suggest that *causal control models* are employed in action control which explicitly represent causal structure involved in action and help to identify control acts that can achieve goal states (Christensen & Bicknell, 2019; Christensen et al., 2015).

It is plausible that cognitive action representations include representations of both action forms and causal structure, but causal representation is crucial for intentional control and flexible problem solving. Intentional control of action characteristically involves producing an action with particular features *because* an action with these features will bring about a goal. The representation of instrumental relations is based on the representation of causal relations. Flexible problem solving involves representing the causal structure of novel problems and finding a means to produce a causal intervention which will bring about a goal state.^5^

We can further illustrate the role of causal control models in action control using the example of braking. A causal control model involved in the control of braking will represent key causal factors such as *braking strength*, *grip*, *speed*, and *braking distance* as distinct, interrelated components of braking. This allows the individual to formulate a wide range of braking strategies, and adopt strategies appropriate to the conditions and their goals. Some of the possible strategies include *early braking*, in which gentle braking is applied far from the point at which halting or a desired speed is attained, and *late braking*, in which a high speed is maintained until relatively close proximity to the target point and speed is rapidly reduced by means of hard braking. A much more advanced example of the use of causal representation in formulating action strategies can be seen in a video lesson by the climber Tomoa Narasaki.^6^ Narasaki is one of the best boulderers in the world, and has a dramatic style which involves frequent use of leaps between climbing positions that are far apart. These moves are called 'dynos'. In this video Narasaki explains his technique for performing a particular kind of dyno. What is of most relevance here is that he gives a detailed rationale for each component of the technique that is based on a deep causal understanding of the technique. This causal representation includes principles that can be used, not just for this particular technique, but for refining other techniques and formulating new strategies.

Our notion of causal control models is related to Pacherie's (Mylopoulos & Pacherie, 2017; Pacherie, 2011) concept of *executable action concepts*. Pacherie (2011) illustrated the idea of executable action concepts by contrasting them with observational action concepts which may not be executable. Thus, a spectator at an ice-skating competition may acquire the concept of a triple-axel by watching it being performed, but is unlikely to be able to perform it themselves. Pacherie argues that, since possession of the observational concept doesn't guarantee the ability to perform the action, in order to possess an executable action concept the individual must *already possess* motor representations capable of producing the movement. Mylopoulos and Pacherie (2017) argue that executable action concepts are executable in virtue of being linked to motor schemas which are acquired through bottom-up learning processes.

A difficulty with this account, however, is that bottom-up motor learning in most cases depends on the action being first produced intentionally. Indeed, it has been a standard assumption that skill learning involves an initial phase in which the action is produced intentionally (Anderson, 1983; Fitts & Posner, 1967). There are possible exceptions in which the structure of the movement is produced incidentally as part a larger action and consolidated by bottom-up learning (Sun et al., 2001). Sequence learning tasks such as the serial reaction time task (SRTT) are designed to exploit this possibility as a means for studying implicit learning. In the SRTT the participant presses buttons in response to cues (Robertson, 2007). They are not informed that the sequence of cues/button presses contains a pattern. On subsequent tests participants are faster at the task, indicating they have some learning of the sequence. It was hoped that tasks like this would reveal purely implicit learning, operationally measured as speeded response combined with lack of explicit awareness of the sequence. However, participants do learn some of the sequence structure explicitly while performing the task and this appears to fully explain speed improvements (Krakauer et al., 2019). Thus, even in tasks specifically designed to elicit implicit, bottom-up learning it has proven difficult to do so. Masters and colleagues have attempted to develop training methods which allow the movement patterns of a skill to be learned largely or entirely implicitly (Masters, 2000; Poolton et al., 2006). However, it has proved difficult to apply these methods to complex skills (e.g. Poolton & Zachry, 2007). For most complex real-world skills like performing a dance step or changing gear in a manual car there is no other practical way to initially generate the action than by intentional control.

Thus, for the most part, the individual must already be able to intentionally produce the action before bottom-up motor learning can start to occur. Bottom-up motor learning refines and consolidates movement patterns that are intentionally produced. It doesn't construct novel movement patterns. Pacherie is right that to develop an executable action concept the individual must already possess motor representations capable of producing (at least an approximation of) the reference movement pattern. But in the initial stages of motor skill acquisition the individual does *not* have an integrated motor representation that is specific to the movement pattern being learned. The individual usually needs to *construct* a *cognitive* representation of the desired movement as an integrated structure assembled from intentionally controllable motor components.

This brings us to a crucial phenomenon that any theory of action and skill must accommodate, namely the ability to intentionally produce novel movement patterns. The basic level of control in intentional action is not the ability to produce 'basic actions', in the philosophical sense, it is the ability to intentionally control movement parameters such as postural parameters, direction, distance, speed, force, and so on. To intentionally produce novel movement patterns as functionally integrated wholes it is necessary to represent causal interdependencies amongst at least some of these parameters, such as between position, distance, speed, time, and force.^7^ Thus, causal control models of the same kind as we described for braking are used in the fundamental control of movement.

Infants and young children learn a repertoire of basic coordinated actions, including pointing, reaching, grasping, manipulating, stepping, and so on. These actions are basic in the sense that they come to function as units which will be employed in the construction of more complex actions. They incorporate stereotyped movement patterns and their control is likely to incorporate linked conceptual and motor schema representations in the way that the Mylopoulos and Pacherie model describes. But they are intentionally *controllable*, in the sense that their parameters can be intentionally adjusted to achieve a variety of goals. To explain this we need to supplement the Mylopoulos and Pacherie model of executable action concepts with the account of causal control models that we are proposing. Typically, in skill acquisition, such as when learning to play a musical instrument, more basic intentionally controlled movement capacities are adapted for the specific demands of the skill. During skill learning cognition leads in the construction of new actions to suit the task demands. Once a novel action structure has been constructed, consolidation and refinement will occur across the whole control system, including the formation of integrated motor schemas *and* the formation of integrated causal control models.

#### Translation between representational systems

Theories of action control face the problem of understanding translation between and within representational systems during action control, including multiple perceptual modalities, visual and verbal linguistic representations, emotion experience, gestures, computer and web interface 'languages', the iconography and signaling conventions of driving on roads, maps, music representational systems such as notation and tab, and so on.^8^ Hierarchical models of intentional control, such as that of Mylopoulos and Pacherie (2018), must explain translation across different levels of abstraction. Translation across all these representational systems and levels plays a central role in problem solving and flexibility. Abstract goals and plans must be interpreted in more concrete situational terms. Flexibility hinges on being able to vary the way actions are performed in relation to features of the situation while realising the features of action critical to achieving the goals. Learning involves abstracting action features from instances in a way that allows varied concrete implementations. Recent philosophical theories of the architecture of action control, such as the DPM and Mesh models, have not so far addressed these issues but there is a long tradition of work on them in other fields (Fitch & Martins, 2014; Lashley, 1951; MacKay, 1982; Ondobaka & Bekkering, 2012).^9^

A connected issue that has received recent attention in philosophy is that of the perspective of the representations involved in action control. Pavese (2019) develops the idea that some representations have a distinctive *practical mode of presentation* or *practical perspective*. She argues that these are imperative representations which specify a method of performing a task in terms of the abilities of a system that can implement the method. She claims that motor commands and motor schemas are examples of this kind of representation. We agree this is an important form of practical perspective, but we need to also understand the form of practical perspective of the representations used in the problem solving by which schemas and motor commands are formulated and evaluated. We'll call this the *perspective of control*. The perspective of control encompasses all of the phases, levels and aspects of control, many of which have their own perspectival characters, including those of distal decision-making and proximal control of execution.

#### The structure of control

In addition to causal control models, previous explications of Mesh have identified two further components of action control: forms of higher order performance and metacognitive awareness. Before describing these in detail, however, it will help to clarify the structure of control. Mesh is similar to the DPM model of Mylopoulos and Pacherie (2018) in depicting action control as involving a hierarchical structure. Mesh has not yet been very specific about the details of the nature of the control involved in the hierarchy, whereas the DPM model, based on Bratman's account of intentions (along with Searle (1983), Brand (1984), and Mele (1992)), specifies a control organisation that involves multiple levels of intentions which are responsible for specific aspects of action control. In particular, a *distal* intention, commonly formed outside the action context, represents the overarching goal of the action. *Proximal* intentions are formed which specify how the distal intention is to be implemented in the context of performance. *Motor* intentions specify the motoric means by which proximal intentions are implemented. Here we extend Mesh by specifying the structure of control in more detail. This account shares with the DPM model the idea that intentional action often involves a hierachical goal structure, but departs from it in certain respects which in part stem from a departure from Bratman's model of intentions.^10^

One way to conceptualise control is as the ability of the agent to achieve its goals. We'll call this the *goal-based* conception of control. Mylopoulos and Pacherie (2018) and Shepherd (2021) employ this conception.^11^ A different way to conceptualise control is as the ability of an agent to solve the problems that it faces. We'll call this the *problem-based* conception of control. Both concepts of control are useful but the problem-based concept is important for understanding adaptive control systems and the structure of control in skill learning. Thus, when we perform a full analysis of a biological control system we need to determine both the proximal goals (the represented goals) and the ultimate goals, which are solutions to adaptive problems faced by the biological agent. These problems are to a significant degree independent of and prior to the explicit goals of the control system. Solving them is often obligatory or very difficult to avoid. The relationship between proximal goals and adaptive problems will often be imperfect, and evolution will generally tend to bring the represented goals of organisms into alignment with their adaptive problems. Proximal criteria used in the control of eating include satisfying hunger and enjoyment of the experiences of eating. The primary adaptive goal is nutrition. Humans can adopt conceptualised nutrition as an explicit goal of eating but they need not. The proximal control criteria for eating can be satisfied while the adaptive problem is not. In cases where conceptualised nutrition is a goal of eating it may correspond imperfectly to objective nutrition. Thus, goal-based and problem-based control can be dissociated.

Humans are a highly social species and are exceptionally flexible in developing varied lifestyles and technologies which have allowed the colonisation of almost every kind of terrestrial environment on earth. This flexibility in lifestyles is founded on an exceptional capacity for flexible skill learning. Human evolution has thus endowed us with skill learning capacities which are extremely good at solving the ecological and social problems we face. *Uncertainty* plays a central role in this flexibility. Humans face a fundamental and pervasive uncertainty concerning their goals. Their goals correspond imperfectly to their problems and they must learn about the structure of the problems that they have. *Problem discovery* thus plays a central role in skill learning. Skill learners typically begin with poor representations of their problems. Their goals correspond imperfectly to their problems and they must learn about the structure of the problems that they have, and learn to form better goals. This learning occurs at every level, from the specific problems involved in performing particular actions up to and including self-conception, whether to engage in the skilled activity at all, and to what degree.

More specifically, uncertainty and problem discovery play a key role in the structure of action evaluation. On a goal-based hierarchical model of control, such as the DPM model, performance at a given level of control is evaluated with respect to the goal at that level and to higher level goals. Thus, the success of motor performance is evaluated with respect to whether it achieves the goal specified by the M-intention, and whether this satisfies the goal specified by the P-intention. However, there are certain phenomena which arise quite commonly during skill learning which don't fit this model very well. An action can go according to plan yet be assessed negatively. For example, an inexperienced guitarist might perform with a band at a gig in a way that they have planned to, and which they consider to be aligned with their norms for playing well, yet later evaluate their performance negatively when they review a recording. This later evaluation may be based on performance norms they had not previously considered, but which are highlighted when they assess their performance from the perspective of a listener and compare it to performances of more advanced players they admire. For example, they might realise that their playing was overly busy, failing to complement the song, and too loud, overshadowing the rest of the band.^12^

An action can also go contrary to plan yet be assessed positively; a mistake which proves to be a 'happy accident'. For instance, you might accidentally shake out more hotsauce on your eggs than intended, yet regard the outcome as superior to the intended quantity of hotsauce.

These possibilities can't be explained if the only evaluative criteria are the goals specified by the intentions.

#### The action evaluation system

To understand these phenomena we need to recognise a broader set of evaluative criteria. While it is often the case that a specific explicit goal operates as a primary focus of action selection and regulation processes, this goal is only one item amongst a complex set of criteria used to evaluate the action. Some criteria are low level and generalised. Thus, all actions are evaluated for efficiency, regardless of whether efficiency is an explicit constituent of the content of the goal of the action. Other criteria are higher level and also generalised. A bluegrass musician will evaluate their playing according to their internalised aesthetic norms for bluegrass music. Some norms are specific to the action type, such as technique criteria. Some norms are specific to an individual, such as a personal playing style. In the performance of any given action an ensemble of criteria will be operative in evaluation processes. Criteria other than the primary goal can be used to evaluate the primary goal and its implementation. These additional criteria are themselves imperfect and subject to learning. A novice has evaluative criteria for the skill which are impoverished and poorly reflect the norms of the skill domain. Experts often have very rich, articulated evaluative criteria. For this reason, instruction and other forms of social feedback can play a vital role in guiding learners. Techniques for self-assessment which use an external perspective, such as recording and analysing performances, are also very valuable because they allow the individual to better apply performance norms they have acquired from an observer's perspective to their own performances.

Thus, we add to Mesh the proposal that skilled action evaluation is based on an *action evaluation system* (AES) which develops during skill learning.^13^ The AES plays a role in the cognitive processes of intention formation and in the control of action execution. Action evaluation is holistic: no single criterion has strict dominance (e.g., there is no strict master goal) and the weighting of criteria can vary across contexts. The breadth and depth of evaluation will vary across contexts, but a complex set of criteria are often operative in the control of action execution.^14^

In this respect the account departs from Bratman's model of intentions. As described above, Bratman's model has a strongly punctate structure in which there is a phase of deliberation which culminates in commitment to an intention, followed by a phase in which cognitive processes are focused on implementation of the intention and evaluation of it is bracketed. Bratman's rationale for the bracketing of intention evaluation is based on cognitive resource limitations: he claims that it is not possible to continuously determine the best course of action at each point in time. But while it is true that it isn't possible to perform a comprehensive analysis of the best course of action at each point in time, this doesn't imply that intention evaluation must have the punctate structure of his model. Bratman recognises that intentions may be reconsidered when stakes are high and 'new information comes in', but he regards this as exceptional. He says that it is reasonable to have a default presumption in favour of plan stability rather than reconsideration. Concerning the circumstances in which reconsideration is reasonable, he writes:

Sometimes the stakes are quite high, and there is an opportunity for calm and careful reconsideration of one's prior plan. It seems plausible to suppose that it is in the long-run interests of an agent occasionally to reconsider what he is up to, given such opportunities for reflection and given that the stakes are high, as long as the resources used in the process of such reconsideration are themselves modest (1987, p. 67).

Our model is very different. It is common to have highly imperfect information and it is hence adaptive to continue to evaluate intentions after they have been adopted, including during action execution. This allows them to be flexibly modified and abandoned as more information is gained and as circumstances change. Here we need to distinguish between evaluation of implementation intentions involved in carrying out a plan and evaluation of the overarching intention. We claim that evaluation occurs at every level. The breadth and depth of evaluation varies, and it is certainly true that there is greater opportunity for deep and wide evaluation before and after performance compared with during, but nevertheless, higher levels of control can be 'in play' during performance. Thus a professional bike rider might, during a race, re-evaluate their ability, re-evaluate their strategy for a particular obstacle, re-evaluate their race strategy, or pacing plan (Christensen & Bicknell, 2019; Sutton & Bicknell, 2020). More broadly, an athlete may re-evaluate their strategy for the season, and might even re-evaluate their commitment to racing at this level. For instance, an older rider near the end of their career might switch during a race from assessing themselves as still being competitive at the highest level to no longer being competitive, and decide on this basis to retire. At the other end of the skill career time line, we can expect that evaluation of commitment to the skill to commonly occur during performance during early phases of acquisition and at key career stages.

The basis for such evaluation is the individual's AES. A well-developed, adaptive AES represents relevant evaluative criteria at various stages of intention formation and action performance.

This model of control differs from the DPM model. In keeping with Bratman's model, on the DPM model distal intentions are ascribed the function of terminating practical reasoning about what to do. Evaluation of success is goal-based and top-down. In contrast, our model places more weight on bottom-up processes in which higher-level goals are revised in response to information gained during action execution. Evaluation of intentions does not necessarily terminate with their adoption. Evaluation of success is not only with respect to achieving the goals specified by intentions. We think the DPM model can be readily amended to accommodate the phenomena we're describing, but these are nonetheless features of control that have high significance because they play important roles in learning and flexibility.

#### Performance and metacognitive awareness

Performance and metacognitive forms of awareness play a key role in action evaluation. In contrast, on Bratman's account reconsideration is based on habits and dispositions, deliberation, or by policies. He thus fails to recognise the importance of such forms of awareness. Confidence, for example, can have performance and metacognitive forms. *Performance confidence* is awareness of the likelihood of action success. *Metacognitive confidence* is awareness of the extent to which sufficient information is available for effective decision-making and control. When both kinds of confidence are high, as they are likely to be when a philosophy professor makes a plan to go to the library to borrow a book, depth of evaluation can be low during decision making and performance. When these forms of confidence are much lower, as they will be when learning a new mountain bike riding technique, depth of evaluation will tend to be higher in all stages of action.

According to Mesh, causal representation contributes to performance and metacognitive awareness. A causal control model represents the causal features of the situation relevant to action decisions and control. In the case of braking this will include causal features such as the nature of the surface, the amount of grip, and the amount of braking force that can be applied. Thus, the causal control model is the basis for awareness of the *performance envelope*, or range of performance states that are available. In this case, awareness of the performance envelope includes awareness of the range of braking forces that can be applied without losing grip. Awareness of the performance envelope serves as a basis for evaluating whether to continue with an action during performance. If a breakdown is likely it may be best to abandon the action. At a more finegrained level, awareness of the performance envelope allows the formulation and modification of action strategies. If grip proves to be unexpectedly high, for instance when using a new type of tire, braking strategies can be modified accordingly. When the individual is *uncertain* they may adopt a conservative strategy and attempt to gather more information (Christensen & Bicknell, 2019). Thus, if they are unsure of how powerful their brakes are, for example if they are on a new or recently serviced bike, they might use early braking or ride at a slower speed as they assess the performance of the brakes. If the individual is *confident* they may operate closer to the edges of the envelope.

#### Social and cultural learning and the degrees of freedom problem

The standard approach to skill is individualistic. When seeking to understand advanced skills the focus is on the autonomous abilities of individual experts. When seeking to understand skill learning the focus is on the processes by which an individual's abilities are transformed from novice to expert. Of course, it is understood that teaching and other forms of social learning play a role in skill learning. It's understood that some skills, such as theatre, dance, music, and team sports, involve collective action. Indeed, there is burgeoning transdisciplinary interest in collaborative experiences of, and influences on, skilled performance (Bicknell & Sutton, 2022). Nevertheless, skill theories treat social phenomena as secondary, or subtopics of skill. Mesh has followed this individualistic orientation (but see Christensen & Sutton, 2018).

Attention to the larger evolutionary and ecological context indicates that we should see individual and social aspects of skill as fundamentally interwoven. Human skills are exceptionally flexible, complex, and are generally acquired and practiced in highly social ways. These associations are not accidental. The flexibility of human skill is founded on a sensorimotor system capable of many 'degrees of freedom', being able to adopt an extremely large number of configurations that can be structured in many ways over time. The diversity and complexity of human skills, in comparison with other species, is possible only because of this underlying potential. But the high dimensionality of the human sensorimotor system, combined with the complexity of many skills, presents difficult problems for learning and control. The learner confronts an extremely large *problem space* in which solutions must be found. The degrees of freedom of the sensorimotor system must be steered in ways that realise solutions (Bernstein, 1996). Learning thus presents extremely difficult search and control problems.

One way that learning is made tractable is to acquire skill progressively, beginning with basic abilities that present relatively simple problems and moving to progressively more complex abilities that build on the simpler abilities (Bryan & Harter, 1899). Another way that learning is made tractable is by making it social. Experts and peers provide models of high quality solutions. Teachers can guide learners through the extended pathways to complex solutions. A skill *community* is able to explore the space of possibilities and solutions far more effectively than a lone individual. When a member of a community discovers a superior solution or other form of valuable skill knowledge, this can be propagated through the community (Goodwin, 1994). Many discoveries can be combined into complex, sophisticated methods.

#### Rival expectations

Based on the preceding discussion we can distinguish between two main contrasting sets of expectations for the situation we are investigating. Based on the classical view we should expect major changes to technique and equipment to be highly disruptive for two reasons. Firstly, automated forms of flexibility should be unable to cope with these kinds of changes because they require qualitatively new sensorimotor patterns. Secondly, cognitive processes should also struggle to cope because they employ general purpose representations and problem solving methods. They are hence poorly suited to skilled action control, and should be overwhelmed by the alterations to the complex relations involved in action production. Mesh yields a different set of expectations. An individual with a significant amount of experience will have developed mechanisms for the cognitive control of action which allow relatively fluent coping based on problem solving. This problem solving will be based on causal representation and an action evaluation system employing a complex set of criteria. Control will be more flexible than depicted by Bratman's model, with continuous evaluation even at higher intentional levels. This problem solving will be imperfect, however, especially in an individual whose technical abilities are fairly basic. Social guidance from an expert can supplement individual problem solving by directing the learner to better solutions.

### The nature of our study

We conducted a field study investigating responses to major changes in a rider with several years mountain biking experience but beginner-level technique.

We employed a researcher-practitioner approach, in which the authors served the dual roles of investigators and participants (see Bicknell, 2021; Downey, 2022; Downey et al., 2015; McIlwain & Sutton, 2014, 2015; Nemani & Thorpe 2016; Olive et al. 2016; Ravn, 2022; Samudra, 2008; Spinney, 2006; Sutton & Bicknell, 2020). This approach brings attention to theoretically and ecologically significant aspects of skilled action in contexts that are difficult to capture in the laboratory, from the armchair, or when the researcher is unfamiliar with the nuances of a particular community of practice. Our experiences were undoubtedly shaped by our theoretical interests, and the evidence should be viewed as exploratory and tentative. Validation of the kinds of phenomena we describe is needed using other methods. These include broader ecological sampling and laboratory investigation. It is especially important that ecological methods be employed which use structured data gathering in close temporal proximity to performance with theoretically naïve participants. But it must be emphasized that all methods have strengths and limitations. The best overall research strategy is to use a large methodological toolkit and seek to buttress each investigative mode with convergent evidence from others.

The two authors of this paper have differing levels of mountain bike expertise. Kath has been a mountain bike rider for over twenty years. She has worked for global cycling media for more than ten years, taught skills clinics, raced domestically and internationally, and has written academic papers and a PhD on the sport. Wayne, in contrast, has very limited mountain bike riding experience. He is not a raw novice; he has extensive experience of recreational and commuting riding on roads and about fifteen years previously he spent about a year mountain biking regularly, riding once or twice a week. This experience included twisty 'singletrails', tracks roughly the width of a foot trail, with rutting and differences in grip due to the type of dirt underneath his wheels (eg. loose and skatey or smoother ‘hardpack’). He had largely avoided more 'technical' trails including features like ‘rock gardens’ and logs. With respect to mountain bike riding he was self-taught and as a result had not acquired some important basic mountain bike riding techniques. As part of research for a previous paper (Christensen et al., 2015), he read a mountain bike instruction book and gained some familiarity with basic mountain bike techniques this way. Kath had also given him some instruction during the study. He had not, however, spent a significant amount of time practicing these techniques or received any further ‘live’ riding instruction.

The fieldwork session was designed to explore and document Wayne’s experience of the trails through an initial ride with no input from Kath, followed by two major changes. After observing Wayne ride an initial series of beginner-intermediate singletrails, Kath provided instruction on core mountain bike riding techniques, which Wayne then attempted to employ. Secondly, after riding the track on his own bike, Wayne then switched to Kath’s bike, which was a more modern and capable design.

In more detail, at the time of the study Wayne’s bike was approximately ten years old, an aluminium ‘cross country’-style hardtail (meaning no rear suspension), with 26″ wheels, hydraulic disc brakes, and basic front suspension in need of a service. Kath’s ‘trail’-style bike was a few months old. The design reflected substantial changes in bike technology, trends and manufacturing materials. These included: larger 29″ wheels with wide 2.4″ tubeless tyres, which roll over obstacles with more ease and traction compared to Wayne’s smaller wheels with narrower tyres; a more stable and relaxed geometry, which adds traction and confidence on climbs and descents; far more sophisticated front and rear suspension for better traction and compliance, and other modern features such as a 1 × 12 drive train (so no shifting is needed with the left hand) and a dropper seat post which allows the rider to press a lever on the handlebars and move the seat downwards so it doesn’t catch on their thighs when they move their body toward the rear of the bike while descending. The brakes were far more powerful, the frame material (carbon) more compliant, and the handlebars substantially wider, again providing enhanced stability and control. Kath and Wayne are a similar height, meaning they both ‘fit’ the same size frame. However, when Wayne switched to Kath’s bike the contact points were adjusted for Wayne, with the seat height being modified and his own pedals used.

The trail used for this investigation, a popular cross-country loop at the Ourimbah trail network in New South Wales, Australia, was chosen on the basis that Wayne could tackle it with reasonable safety, with guidance from Kath, but which contained obstacles that were more challenging than Wayne's prior experience made him comfortable with. During the ride, Kath gave Wayne the kind of instruction and induction into the mountain bike subculture that would be characteristic of an experienced rider taking a friend on a ride for the first or second time. For example, when Wayne was concerned about riding over a log obstacle she explained and demonstrated key body movements and the amount of speed needed to do it safely, but also encouraged him to walk the obstacle and try it again later if he preferred.

We took photos and recorded video of Wayne’s successive attempts at challenging obstacles on both bikes, and recorded video of Wayne describing his immediate responses to these experiences. We met the next day to write notes on the experience.

## Riding the Ourimbah cross-country track

There were two aspects that stood out as especially noteworthy in Wayne’s experience: a change in technique that dramatically improved descending steeper trails, and adaptations to the increased performance capabilities provided by the second bike, particularly due to its stability over rough terrain.

At the beginning of our fieldwork session, Wayne rode a short loop of ‘singletrails’ without instruction or interference from Kath, who rode behind him, watching and observing. These trails included several relatively steep descending sections of trail, linked together by narrow, winding, rocky connections and the occasional smooth section for the rider to relax and catch their breath. (Understandably) Wayne was riding somewhat nervously and cautiously. He felt that his approach to riding these trails was reasonable given his limited overall experience and that he had not ridden a mountain bike trail in a number of years. Riding behind him, however, Kath could identify specific technical problems. She could see and hear the rear wheel skidding and noticed that Wayne’s body position (and centre of gravity) was quite far forward on the bike. This puts a lot of pressure on the front wheel, which causes several problems while descending, the most severe of which is that it increases the risk of flipping over the front of the bike. In the mountain biking subculture this is referred to as an ‘OTB’ (over the bars)—the standardization of the term suggesting it is a fairly common experience but one to be wary of.

We paused on a long flat section of fire road to discuss the experience of the first section of trails. Worried that Wayne was going to injure himself, Kath provided instructions that would help him ride the section more smoothly and safely on the next attempt. She explained a technique for descending steep obstacles, which involves keeping the feet balanced evenly on the pedals while the rider moves their bodyweight rearward on the bike to maintain balance and stability. She explained this to Wayne verbally, along with an arm gesture showing the effect of weight on the bike in relation to the terrain. She used the cue words ‘butt back’ and ‘weight back’ as a shorthand way to emphasize and direct this technique after the initial description of what to do. Taking advantage of the less threatening and challenging terrain provided by the wide, flat fire road, Kath showed Wayne an exercise which encouraged him to experiment with how far he could move his body rearwards while maintaining momentum on the bike. This involved riding in a straight line at a moderate speed, moving his butt behind the saddle and allowing it to graze the rear tyre. This exercise was designed to increase his awareness of how much space he had to move rearward on the bike, what this felt like in practice, and how this sensation differed to what Wayne thought was the near-maximum amount he could get his bodyweight behind the bike.^15^ The sound and feel of grazing the tire provide aural and kinaesthetic cues that signify the rider has succeeded in the task when it is not possible to see, visually, how far back they have reached.

We then rode the same short sequence of trails again. Wayne now focused on getting behind the saddle during any steep downward sections of trail. The result was a dramatic improvement in the controllability of the bike. This transformed the experience of sustained descents and short, sharp drops or ‘rolldowns’. Rather than feeling threatening, the experience of riding these obstacles felt relatively comfortable—it felt less steep, less rough, less like he was about to have a crash. After applying the technique cautiously to a rocky ‘stepdown’, Wayne immediately began to apply the same technique to other steep, downward sections of the trails: log rolls, steep downward corners, rocky rolldowns, anything where the front of the bike is much lower than the rear of the bike while riding a trail obstacle.

After a longer lap of the trail on Wayne’s bike, Kath gave him her own bike to use for the second lap. She had another bike waiting in the car to facilitate this exercise. The technological differences described above between Kath’s bike and Wayne’s older model bike indicate that Kath’s bike would be more stable, compliant, absorbent and confidence inspiring. While this makes sense on paper, Wayne was nevertheless astonished at how much more capable the bike was in practice. The most immediately striking feature of the bike to him was its greater stability, and the fact that this allowed much better low speed control than his own bike. In comparison, the relative instability of Wayne’s own bike meant it would often feel like it was going to tip over at slow speeds.

The combination of greatly enhanced stability and compliance had a dramatic effect on Wayne’s experience riding the trail. He was able to ride faster on bumpier sections, with the bike soaking up rocks and gaps between them that would produce strong jarring on his own bike. Within 10–20 min of riding this bike his confidence greatly increased. Many descents and ascents he had struggled on while riding a lap of the trails on his own bike—rooty straights, rocky windy uphills, small rocky step-ups, uphill corners, rutted entries into corners, descents littered with a messy array of small obstacles—were experienced as being much more ‘ridable’ than they had been earlier that same day. The bike maintained traction more easily allowing Wayne to pedal and guide it through the obstacle with more control, confidence and ease. He continued to approach some obstacles cautiously, however, and Kath spent some time teaching him to ride over a log, which looked difficult to him but in fact was not. His main difficulty was simply overcoming his fear of the obstacle so that his approach was fast enough to carry him over the rocky ramp that led to the log itself. We make no claims that Wayne was able to ride all sections of the trail, or ride flawlessly ever after. He subsequently crashed when he misjudged a rocky section later in the day, bruising his ankle and wrist and acquiring some grazing.

## Finding theory in action

In this section we use the theoretical concepts introduced in Sect. 2.2 to characterise the processes by which Wayne adjusted to the new technique and the new bike. Wayne was familiar with these concepts, which makes it easy for him to describe his riding experiences in these terms. This familiarity also undoubtedly influenced Wayne's experiences during our field study. However, based on Kath’s extensive experience teaching skills clinics, and riding with mountain bikers at a diverse range of skillsets, we think that the kinds of problem solving Wayne engaged in are not unusual. In particular, his experiences of identifying control problems and experimenting with solutions were in many respects fairly typical for a beginner rider.

### Simple forms of adaptation

Wayne adapted rapidly to the new technique and new bike. Some of this adaptation involved relatively simple forms of flexibility. Simple calibration changes in important parameters, such as braking forces and braking timing, played a role. Both of the major changes improved stability, which had the effect of making feedback control more tolerant or less 'twitchy'. Feedback control processes quickly recalibrated for the new tolerances. As riding became more manageable Wayne could relax more, and reduced bodily stiffness is likely to have made control easier; bumps and other perturbations which might have been jarring and disruptive would now be absorbed more effectively through his limbs. Experience selectively highlighted key parameters, allowing control to be more focused.

### Problem solving using causal knowledge and metacognition

Nevertheless, the changes in technique and bike were large enough to require adjustments by means of strategies formulated using problem solving. Wayne's ability to adapt to large changes hinged critically on an ability to formulate and implement new action strategies 'on the fly'. We can illustrate this by describing in detail a change in riding strategy associated with the technique change of getting behind the saddle. Although Kath wasn’t aware of it, the rear wheel skidding that she observed during Wayne’s initial ride was partly the result of a deliberate braking strategy that he thinks of as ‘tail dragging’, which involves using primarily the rear brake. After adopting the behind-the-saddle technique Wayne switched to an equalized front-rear braking strategy. Wayne was familiar with rear-wheel-based braking from riding as a child, with this experience including riding ‘back pedal’ brake bikes and with using the rear brake to skid out the back wheel on loose surfaces when coming to a halt, a satisfying and popular maneuver. He knew, however, that equalized braking is regarded as the superior technique and he had generally used it in his previous mountain bike riding. He nevertheless initially used rear-biased braking as an improvised strategy in response to control problems that he was experiencing. Wayne wasn’t aware that his weight was too far forward, but he was aware of some of the consequences of this. The load on the front wheel caused instability and had the potential to cause the front wheel to lose grip and slide out, resulting in a crash (which Wayne thought of as a ‘washout’).^16^ Wayne felt that the wheel was most likely to lose grip under braking and using a rear-biased braking strategy helped to reduce this risk.

Thus, causal awareness played a key role in the adoption of the tail-dragging strategy and the later switch to equalized braking. Here we should note that Wayne's perception of the most immediate and important risk that he faced differed from Kath's assessment. Wayne was preoccupied with the danger of a washout due to heavy front braking, whereas Kath viewed the primary risk in Wayne's riding during this phase as being a front wheel washout or OTB crash caused by insufficient rearward weight. Other riders in a similar situation might have interpreted their risks differently and adopted different strategies. Other strategies which reduce the risk of loss of control when riding a difficult descent include putting one or both feet on the ground and scooting down, using both brakes fairly heavily and ‘inching’ down the obstacle (generally ill-advised), avoiding braking all together and focusing on body position, balance and looking ahead to the exit of the obstacle, or getting off the bike and walking (or sliding) the bike down the obstacle.

Metacognition can also be seen in this example. While Wayne thought there was a danger of the front wheel sliding out, he didn’t know in detail in what conditions this could occur. He was still adjusting to the ‘feel’ of the bike on the terrain and was uncertain about the amount of grip available and the braking forces that could be used. That is to say, Wayne was aware that he lacked sufficient information for good control. Estimating these action parameters is complicated by the fact that they are strongly affected by the nature of the surface, which was variable, and by the fact that in a washout the loss of grip tends to be abrupt. In the face of this uncertainty, tail dragging combined with low speed was a relatively safe, conservative strategy. And it worked! Wayne did manage to ride these difficult sections of trail without crashing. Kath’s intervention was to help Wayne ride them more smoothly, more safely and, ultimately, more enjoyably.

Metacognition also influenced Wayne’s use of tail dragging in another way. Tail dragging is a simple strategy to employ because there is no need to precisely coordinate front and rear braking pressures. Wayne was experiencing high cognitive load because he needed to pick a line with care over the deep rutting of the trail to ensure that the front wheel did not glance off the side of a rut and get channeled down it, resulting in a crash. In addition, Wayne was experiencing significant jarring through the handlebars, and he was concerned that if he hit a bump while braking he might accidentally grab the front brake too hard. Tail dragging simplified the cognitive demands of braking and allowed him to direct more attention to line choice. That is, the choice of strategy was based in part on awareness of excessive cognitive load and the need to reduce this load.

One of the main problems with tail dragging is that it reduces effective braking power because braking force is provided by only one wheel, and because it often results in the rear wheel skidding. This in turn means that speed must be kept low. Partly for this reason Wayne maintained a fairly low speed during the descents, but he preferred to ride at a relatively low speed in any case to allow more time for line choice and to minimize the consequences of a crash. He thus didn’t regard the speed limitations of tail dragging as a reason to avoid it in this context.

However, after Wayne began getting fully behind the seat while descending he switched to equalized braking. This was because the control problems that prompted the tail dragging strategy had been largely eliminated. Independently of any detailed causal understanding, the rearward riding posture leads to several changes in the feel and handling of the bike which provide greater sense of control on steep sections of trail. Cues indicating instability are reduced and handling is improved. The arms are more extended, which reduces unwanted side-to-side rotation of the handlebars and, consequently, the front wheel (compared to the freedom of movement that comes with a larger bend at the elbows). But Wayne was more specifically aware that with his weight now towards the rear there was a greatly reduced risk that the front wheel would lock up under braking. The risk of an over-the-bars crash was also much lower. As noted, this danger had not been at the forefront of Wayne's mind but he was aware of it (he had experienced such a crash previously). Now that his weight was positioned rearwards, and the bike could rotate forwards without pitching him forwards, he became aware that an OTB crash was a lot less likely.

Indeed, the front wheel could now rise and fall much more easily as it tracked over obstacles. This made line choice less critical because there was less chance that the front wheel would glance sideways when it struck the side of a rut. This reduced cognitive load. There was less jarring through the handlebars, making it easier to judge and execute braking pressures. With braking distributed between front and rear, overall grip was increased and there was less chance of either wheel skidding. Wayne became more confident about applying much stronger braking pressures than he had previously. And since the improved handling made similar riding problems more tractable, he became more generally confident about tackling various kinds of descent obstacles. There were distinct limits to these improvements, however, and there were some descents that he still regarded as too challenging. For these he would dismount and walk.

We can illustrate changes in strategy in response to the new bike with the example of a decision to tackle a particular ascent. It was short, relatively steep and had a somewhat loose surface. Wayne tackled it several times on his own bike and once on Kath’s. On his own bike Wayne found the ascent challenging because he needed to begin with high momentum in order to climb it. There were two problems that contributed to this. One was that he had relatively little grip because of the geometry of his bike and the tires. Specifically, on this slope, with its loose surface, if his speed became too slow while using high power pedal strokes the rear wheel could lose traction and 'spin out'. The other was that his riding position on this bike had a relatively high and forward center of gravity, which meant that the bike felt unstable and 'tippy' when riding at slow speeds. If Wayne was going too slow he needed to come to a complete halt and dismount, or he would fall over. The approach to the ascent was downhill, and each time he made the approach he needed to quickly decide whether he had the right line and was going fast enough to make the ascent successfully. He made it up the first time on his bike but stopped on the second attempt because he didn’t think he was going fast enough.

On Kath’s bike Wayne decided to tackle the ascent even though his approach was slow. This point is worth emphasizing because it highlights the way that the different capacities of the new bike led him to use altered riding strategies for obstacles that he had not yet experienced on the bike. Had Wayne been on his own bike he would not have attempted the ascent with the approach that he had at this point. He did attempt the climb because he was confident that the low speed stability of the bike and its grip would allow him to ride it slowly, with less risk of falling over and less risk of losing traction. This proved to be the case. He found that he could come to a near halt during the climb without falling over, and the increased grip of the tires meant that he could use slow, high power pedal strokes without the rear wheel spinning out.

To sum up, Wayne was able to construct riding strategies ‘on the fly’ based on causal and metacognitive awareness. He could form, evaluate and modify strategies based on awareness of factors such as instability and threatened loss of grip. The strategies could take into account multiple factors, reflecting an awareness of how causal factors interrelate in riding. Wayne also selected and adjusted strategies based on sensed uncertainty and risk. This problem solving ability extended to large changes in causal relations associated with major changes in technique and equipment, and hence allowed him to cope with these changes.

It's important to note that Wayne's adaptations went beyond the formulation of specific strategies for particular problems. Wayne showed generalised learning in the sense that each major change allowed him to solve new classes of control problems. As he formulated and implemented new strategies he was also learning about the underlying causal structure of control. He was, thus, extending his causal control model as well as refining it.

### Difficulties in adaptation

Difficulties and limitations in Wayne's adjustments are also revealing.

The new bike had only a rear derailleur rather than front and rear. This simplified changing gears but Wayne had well-entrenched gear changing methods which involved coordinated shifting of front and rear derailleurs. It's worth emphasising just how important gear changing is in mountain bike riding. With frequent, rapid changes in gradient and other trail features, it's necessary to change gears often. Smooth, fast riding depends on anticipative gear shifts, especially when the change in gear is large. When encountering a steep slope, for instance, the rider may need to shift from a high to a low gear, and be in the right gear to effectively apply power as speed slows. It's desirable to maintain as much speed and momentum as possible. Wayne's technique for such a situation involved making several shifts in sequence. An initial anticipative shift selects the middle or small front chain ring (lower range gears) and a rear gear that is medium-to-low but high enough to 'catch' the initial phase of slow-down and extend the speed and momentum. Multiple subsequent shifts downward are then made, using the rear derailleur, as slow-down continues, until the right gear for sustained climb is reached. Selecting the wrong gear for a shift disrupts the smooth progression. When the gear is too high or too low the rider will 'bog down' or spin, and either way lose speed and momentum. A further consideration on Wayne's bike was that his gears would sometimes not shift under heavy load, making it important to shift before high power output was required. This was not the case with Kath's bike, which shifted smoothly during high-power pedaling on climbs.

On Kath's bike Wayne had to inhibit his urge to operate the front gear system and reorganise the way that he made anticipative gear shifts. This required heightened attention. An especially attention-drawing feature of the alteration was that in the location where Wayne would operate his front derailleur there was a lever to activate the 'dropper' post. This, in combination with pressure (or lack of) on the saddle, lowered and raised the seat. Lowering the seat during descents gives more freedom to move backwards and forwards as needed. But having the seat drop is not something which the rider will want to happen unexpectedly when trying to change gears or pedal up a hill. When raised the seat would spring upwards to its normal position, and was, in effect, a spring-loaded piston driving towards the rider's crotch. This bike feature was unlike any that Wayne had previously experienced and he found it somewhat disconcerting.

Wayne was able to modify his gear change method and learn to use the dropper post, but these adjustments were more effortful and less smooth than those described in the previous section. Why this should be so raises interesting questions. In general, it's reasonable to expect that some modifications to control are easier to make than others because the control system is better prepared to handle some kinds of change than others. Piaget's (2015) distinction between assimilation and accommodation is one expression of this idea. In the Piagetian picture increasingly powerful/flexible forms of problem solving ability develop in a progressive sequence as more abstract/deep concepts are learned. The *Einstellung* (Luchins, 1942) and *functional fixedness* effects (Duncker, 1945) are manifestations of the somewhat contrary-seeming phenomenon of increases in rigidity with learning. There is no deep conflict, however. Learning can involve increases in rigidity with respect to some aspects of control together with increases in flexibility with respect to others.

We can develop a preliminary explanation for differences in difficulty in this case which draws on the resources developed in Sect. 2.2. With respect to the new bike, changes in attributes such as stability and grip were relatively easy for Wayne to incorporate into his riding in at least an initial, basic way. This may be because, although the parameter *values* were substantially different to his own bike, the parameters themselves, and their role in control, were reasonably familiar. He could therefore adjust his existing methods relatively smoothly. But other differences involved more substantial changes in causal relations and more extensive changes in control operations. Thus, a familiar operation needed to be 'remapped' to a different mechanism with drastically different causal effects, along lines such as {[L-LEVER-OP → F-GEAR-OP] ⇒ [L-LEVER-OP → SEAT-OP]}. Since the operations involved considerable novel structure, the structure needed to be composed in working memory, with implementation and monitoring requiring greater attention than more familiar control operations.

More generally, based on the causal control model account we could expect that skill learning will often exhibit a somewhat Piagetian pattern of increases in generalisation and flexibility which arise as the learner learns to solve varied causal problems. More generalised causal representations develop which capture deeper structure, and more powerful and flexible forms of control develop in order to efficiently manage varied problems.

An even stronger limitation in Wayne's ability to solve the riding problems he was facing is evident in the fact that he needed instruction on the correct implementation of the behind-the-seat technique. During the initial ride he was aware that he was experiencing control problems and rode cautiously for this reason. But he was unable to diagnose the source of these problems to specific technical flaws. At this point he assumed that he simply needed more experience in order to improve calibration and refinement, as opposed to making large technical changes.

This failure in problem solving is all the more striking because he understood the technique abstractly and believed he *was* implementing it. When Kath explained verbally the technique of getting behind the saddle during descents, the information was already familiar. He had not known of the technique when he was mountain bike riding by himself many years previously, but he had since learned of it from a mountain bike instruction book. He knew that good riding technique involves shifting one’s weight backwards during a descent to maintain even weight distribution across both wheels. What he didn’t realize is that he was implementing this technique incorrectly. More specifically, he didn’t realize that he wasn’t moving nearly as far backwards as he could and should. From his perspective it seemed like he was moving backwards to about the limits of rearward movement for his body. This was far from being the case.

To understand how Wayne could be as mistaken as he was about this it will help to note that in riding on roads—which was the bulk of his riding experience—there is relatively little need for front-rear body movement. Consequently, a relatively small amount of rearwards movement felt like a lot. Moreover, although Wayne knew that it was important to maintain even weight distribution across the front and rear wheels, he was not used to maintaining this form of awareness and had been preoccupied by line choice. There is a distinctive ‘feel’ to a weight distribution that is too far forward in a descent, which notably involves pressure on the hands and wrists. Wayne had not yet learned to efficiently identify this and respond appropriately.

Thus, although Wayne was able to detect the front wheel instability and formulate a compensatory strategy, he failed to autonomously find a much more effective strategy. This stemmed from a poor representation of weight and balance and a poor awareness of his ability to adjust balance. He failed to properly relate the instability to a forward weight distribution and solve this by moving far enough rearwards. This is despite the fact that he knew the correct technique abstractly. A poor on-the-bike representation of balance contributed to a failure to properly interpret the abstract instructions.

We can interpret these points in terms of the concepts of causal control models, action evaluation systems, and problem discovery. Wayne experienced cues to poor control in his initial ride which prompted him to ride cautiously. But his ability to represent the causes of these problems was underdeveloped and so, while he found a solution that achieved the goal of riding the obstacle, he failed to find a more optimal (smoother, safer, speedier) solution. Once he had learned the superior technique his causal control model was altered and his capacity for action evaluation improved. He became aware of an expanded range of body movements and as he experimented with this range he gained new information about the interrelations between weight distribution, stability and handling. He could now interpret high pressure through the wrists as a sign of weight being too far forward. He could better interpret perceptual cues related to balance and perform bodily adjustments to modify weight distribution more appropriately. Putting this in more general terms, he had acquired a revised understanding of balance control on the bike which yielded a generalised improvement in his ability to solve riding problems.

### The problem of translating between representational systems

The difficulties Wayne experienced involved a failure to properly translate between abstract and situated representational systems. As such, they help to reveal how these mappings are constructed. In the earliest stages of skill learning the individual must laboriously construct concrete interpretations of abstract action descriptions. This is hampered by two factors. Firstly, the individual lacks systematic representations of skill-specific phenomena at the level of concrete control of execution. Secondly, the individual lacks well-developed systematic mappings from abstract to concrete representations. In this case Wayne lacked a fully-developed systematic representation of the range of positions he could take on the bike and their relations to balance. Once he had learned to move his body backwards, and experienced the technique in an approximation of its correct form, he developed an awareness of balance and stability which he could relate to his abstract knowledge of the structure of the technique. He had thus developed a representation of the structure of the technique from the perspective of control which he could use for control. One way to describe this is that he had now acquired a relatively well-structured executable action concept for the technique. However, much more practice would be needed to consolidate this concept in relation to a well-developed causal control model for implementation.

### Improvements in the structure of control

Wayne experienced a significant degree of uncertainty throughout the ride. His intentions, in particular the riding strategies he adopted, involved commitment that was always qualified and evaluated during performance. He maintained awareness of opportunities to abort actions and he did so on several occasions, such as the one described above where he initiated an ascent but stopped part way for fear of losing traction at a higher section of the ascent and falling over. He modified strategies both prior to and during execution to reduce problems, increase the chances of success, and, later, to exploit improved capabilities. His intentions were thus much more labile than Bratman's model recognises. This lability was based on an action evaluation system which could evaluate intentions against a complex ensemble of further criteria represented by the AES. Indeed, the learning process hinged on this.

Wayne's uncertainty was especially high in the initial stages of the ride. He was unsure of which trail sections he could and could not ride safely and he was unsure of whether his riding strategies would be effective in negotiating obstacles and avoiding crashes. His ability to evaluate his performance was also limited, as evident in his flawed diagnosis of the stability problems he was experiencing. Thus, his control ability was relatively poor in both the goal-based and problem-based senses. His ability to achieve the goals that he had was modest and not reliable enough to provide reasonable confidence. But he was also uncertain about his goals, and not able to form all of the right goals, because he lacked a good understanding of his riding problems.

Wayne’s control ability improved over the course of the ride in both the goal-based and problem-based senses. He became better able to achieve the goals that he had, and he became better able to solve the riding problems that he faced. This was based in part on improvements in his understanding of his riding problems, which included greater ability to evaluate action strategies and performance, and form appropriate goals. Improvements in evaluation ability, with enhanced ability to manage uncertainty, are critical to mountain biking, which routinely involves riding unfamiliar, challenging trails.

The technical improvements in particular involved a relatively deep form of problem discovery. As Wayne learned how to properly implement the behind-the-seat technique he was learning both technique-specific and more generalised representations. He was refining a technique-specific concept linked to a causal control model for the technique. These representations incorporated more generalised representations, such as of body position on the bike, balance state, terrain, grip, speed, and so on. These more generalised representations allowed improved representation of a larger set of riding problems. This enabled the formulation of an expanded range of riding strategies which were more effective. These representations also improved the ability to interrelate techniques and strategies by means of common features. Thus, when one strategy is succeeded by another, such as a descent followed by the negotiation of a corner, features of each can be related to each other and adjusted to provide good fit. The speed and line of the descent can be shaped to set up a good entry to the corner and an efficient cornering line, for instance. Action-specific representations are thus integrated into a global *state-space of control* which represents the situation, performance state, and action possibilities. This state-space can be more or less well integrated, and the more integrated it is, the better able the individual will be to produce coherent complex action.

### Social and cultural influences

Wayne's prior mountain bike riding experience was not insignificant, involving about a year of riding one or more times a week on a mixed set of singletrack and fire trails. This provided him with enough skill to engage in the problem-solving described above. But his learning was based largely on solo discovery, adapting skills from road riding and casual BMX-style riding in childhood. The kind of flaws in we’ve described in Wayne's technique, and the limitations in his problem solving ability, are common in individuals who attempt to teach themselves complex skills. Rich engagement with teachers and a skill community can scaffold skill development, allowing the individual to develop solutions and an expanded sense of what is possible (Aggerholm & Hølbjerre-Larsen, 2017).

One of the most important ways that a skill community can guide individual learning is by furnishing skill norms. As we described above, during the initial ride Wayne was aware that he was experiencing control problems but thought his riding was reasonable given his experience and the context. This is an example of a general phenomenon: learners tend to possess impoverished norms for the skill domain, and this hampers their ability to evaluate and improve their performance. In this case, Wayne did not have a good grasp of what kind of performance he should be able to achieve, given his base skill level. In fact, at that point he could relatively easily achieve a much higher level of performance. In general, it is difficult for learners to know what performance standards could be expected for their level and experience. This in turn limits their ability to diagnose problems in their methods.

Skill communities often have highly developed performance norms which orient individuals. In mountain biking, speed is a highly valued performance norm. Wayne was not especially concerned with speed at this point, and Kath found it amusing that Wayne’s initial reactions to the new bike were primarily focused on its improved low speed handling. Speed, though, is only one element of a set of norms for assessing quality of performance. Smoothness and efficiency are also valued. These attributes are integrated into an umbrella concept of *flow*,^17^ which serves as a goal for riders and trail builders (Bicknell, 2016). Other norms concern safety and risk management. In this respect, a feature of the risk norms for mountain biking that is striking to Wayne is the acceptance that there is a fairly high level of risk that is ineliminable and must simply be accepted. Crashes and injuries are simply part of mountain biking. Thus, through social interaction learners acquire concepts for normatively characterising performance which help them to evaluate their own performances and set goals. Group riding, both social and competition, exposes the individual directly to the performance abilities and conventions of others, providing further information for self-evaluation and goal setting.

The ability to use social information is itself skill-dependent. For example, in riding with Kath, Wayne was aware that she was a more capable rider. However, from this he gained little information that was useful for his own riding. Because Kath was so much more advanced, her performance ability did not serve as a useful benchmark for him and he was unable to identify the structure of her methods in a way that would allow him to copy them. Individuals with more advanced skill are often much better at identifying the structure of methods used by others (Bicknell, 2010, 2011). This can allow them to copy and adapt them for themselves, or identify problems which should be avoided or corrected. Teaching can scaffold learners through this limitation. The learner's limited action evaluation system is supplemented by the much more sophisticated action evaluation system of the teacher. In such situations, the causal control model of the more experienced rider can help to develop the causal control model of the learner.

**Fig. 1:**
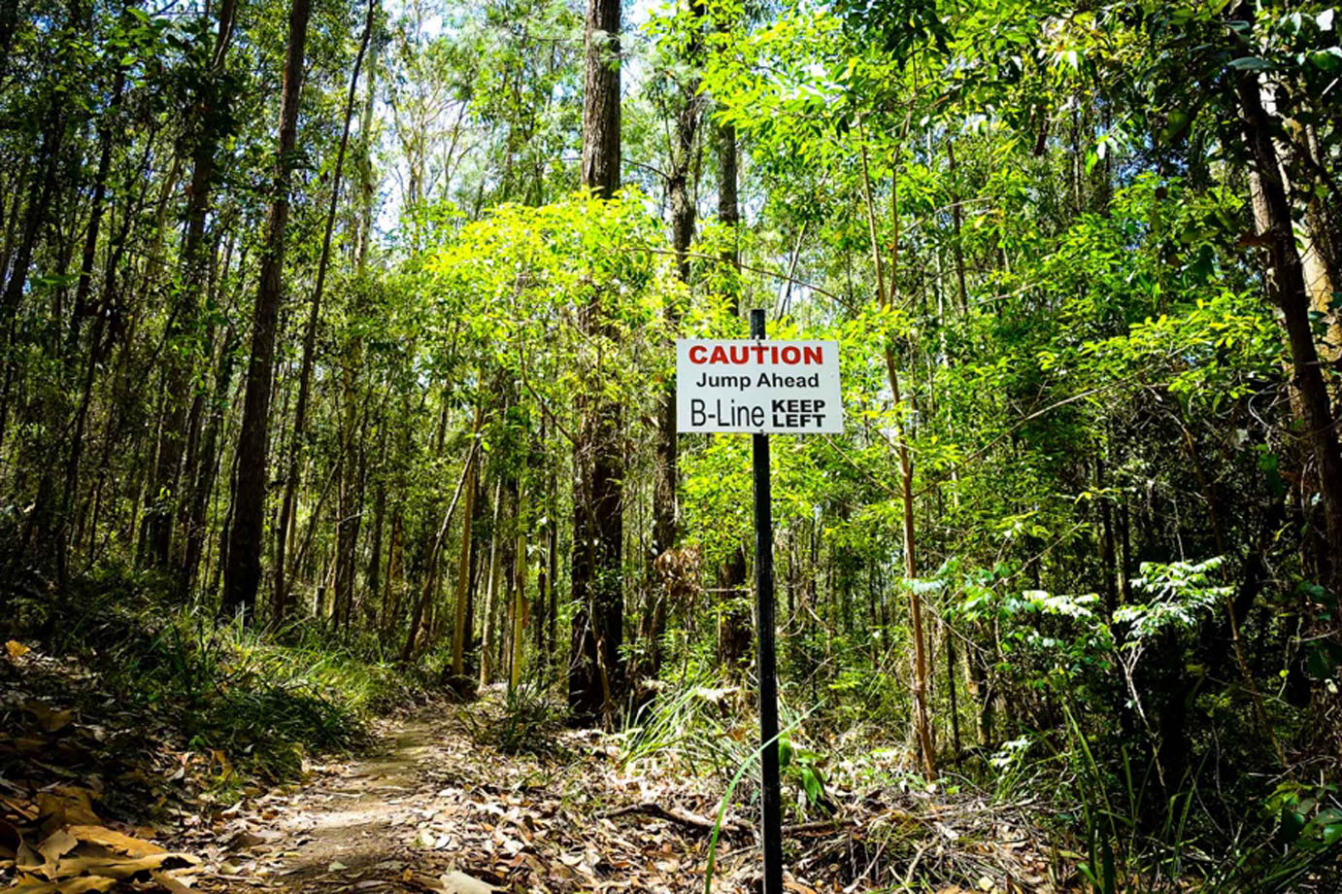
Trail sign, Ourimbah cross-country mountain bike track. This sign prompts riders to prepare for the jump line on the right, or to veer left to avoid it. Importantly, it is placed on an unremarkable section of track, at eye height, to catch the rider’s attention with enough time to make a decision about how to approach the upcoming section of trail, without having to stop riding in order to do so. Photo by Kath Bicknell.

The mountain bike community routinely scaffolds the experiences of the individual in other ways, too. The difficulty and type of experience that riders have is shaped by trail and bike design. Trail grading gives riders information about the difficulty of particular trails which allows riders to decide in advance whether a trail will be within their ability. Grading systems like this also provide individuals with benchmarks for assessing performance. If an individual finds an intermediate trail difficult they are able to locate themselves within the spectrum of abilities within the community. This can in turn guide goal setting for learning and performance. An individual having difficulties at a particular level can seek to identify technical limitations that are holding them back, and work on those, for example. At a more immediate level, signage on trails alerts riders to the required skill level needed to ride specific, upcoming obstacles. Double- or triple-downward arrows before a particularly steep section of trail that isn’t clearly visible on approach serve this function on some trails, alerting the rider to challenging terrain they typically cannot see until they are already riding it. In other trail communities a sign saying ‘warning’ may be used. An alternative convention is signs pointing to A-, B- or C-lines, indicating the technical difficulty of upcoming sections of trail and encouraging riders to make a decision about which line to take (Fig. 1).

Indeed, the mountain bike community has a rich set of community-specific caretaking practices which manage awareness, decision making and problem solving. Additional practices include:

- ‘Pre-riding’ (looking at a trail slowly, with an explorative mindset, before riding it at speed);
- ‘Sessioning an obstacle’ (stopping to look at and practice an obstacle before riding it at speed);
- ‘Riding and scoping’ (eg. riding around a jump while looking at it sideways during the first run of a trail and deciding whether it is safe to attempt on the next lap of that section of trail);
- Building up ‘reserve techniques’ (which can help to regain control if the speed, shape or pitch of the bike mid-obstacle indicates a crash is imminent).

Communication methods are also used to manage problem solving. For example, the question, ‘Is it rollable?’ is one that a skilled rider may ask another before attempting a trail for the first time. If a trail is ‘rollable’ it means there are no gaps that need to be jumped—as long as balance and momentum are maintained everything is ‘rideable’. The trail may well be frighteningly steep and require a high skillset in a number of other areas, but it will be manageable for a rider with a particular set of abilities.

To sum up, skill communities scaffold learning and performance in many ways, allowing higher levels of performance to be achieved and a better, safer quality of experience. The practices which achieve this do so to a significant degree by enhancing the decision-making and problem solving of the individual. Indeed, the engaging nature of sports like mountain biking rests on achieving a complex balance between approachability, challenge, and safety. Mountain biking has been very successful in this respect, and is a fast-growing sport (eg. Latz, 2020). This point is worth emphasising because it helps make the case that the phenomena we’ve been describing are not marginal or unimportant—they’re integral to many skills and can be crucial to their success.

## Conclusion

The ability to formulate action strategies and control their execution is a central issue for understanding action and skill, yet there is very little work on it. Here we found that even an individual with relatively modest skill experience can be capable of fairly complex, fast-paced construction and control of action strategies. Our results need to be validated by further ecological and laboratory-based investigation but we are confident that the core phenomena we've described are real, and that the use of strategies and problem solving is very common in skilled action. The kind of problem solving we found, together with its flaws, is likely to be fairly typical for individuals in relatively early stages of skill learning. But in skills which require significant levels of flexibility—such as mountain biking and climbing—problem solving is also likely to be central to the most advanced levels of skill. It is consequently of high importance that we develop a better understanding of the mechanisms which support these abilities and the way they develop during skill learning. We've argued that causal representation, performance awareness, metacognitive awareness and action evaluation all play important roles and operate together in a complex, integrated action control system. Our account of these mechanisms goes beyond previous work and adds to the Mesh theory of skill a more detailed model of action control.

## Footnotes

1 See Preston (2013) for a related discussion of Bratman’s account.

2 See Chisholm (2008) for an in-depth exploration of the relationship between bodily characteristics, technique and environment in climbing.

3 This video shows six different strategies used to solve a particular problem at the 2021 World Championships: https://www.instagram.com/p/CT9pAfTpMmm/.

4 A video of squirrels engaging in this kind of problem solving can be seen here: https://theconversation.com/we-used-peanuts-and-a-climbing-wall-to-learn-how-squirrels-judge-their-leaps-so-successfully-and-how-their-skills-could-inspire-more-nimble-robots-165524.

5 Nanay (2020) and Fridland (2021) argue that action control is based on imagistic representation, drawing on evidence that practice using mental imagery can be highly beneficial. Causal control models should be differentiated from mental images. Imagistic representations do not represent causal relations per se, although causal representation can be incorporated into imagistic representations. Nor is causal representation necessarily imagistic. It is the representation of causal relations specifically that plays a foundational enabling role in the intentional control of action.

6 https://www.youtube.com/watch?v=IqsNJv2VROs.

7 Not all parameter relations need be represented as causally related since the motor system can sometimes ‘fill-in’ some parameters when given others as explicit goals. Indeed, because of the limits of attention capacity learners must often rely on some degree of ‘filling-in’ because they can’t continuously attend to the full structure of the technique for a novel action while performing it. For instance, when learning a new chord the learner might initially attend primarily to landing finger locations then switch to correcting overall hand posture, while largely ignoring timing. But these kinds of cases illustrate limitations of automated ‘filling-in’ because the filled-in parameters will often not correspond to good technique. Focusing on finger locations while neglecting hand posture when learning a new chord is likely to result in poor posture. Once an individual has developed a high level of skill automated filling-in will tend to better correspond to good technique, but this is because the techniques have already been intentionally learned. We thank a referee for prompting this clarification.

8 We’re using the term ‘translation’ in a broad sense which involves the construction of alternative representations of the same, similar or related content (see Christensen, 2020). This includes translation between alternative expressions within a representational system such as a language. An alternative approach would be to restrict ‘translation’ to content re-representations and interpretations across representational systems with distinct formats and use an alternative concept such as ‘mapping’ for such content relating processes within a representational system, such as between levels of abstraction (we thank a reviewer for this suggestion). This raises a complex set of issues. We agree that there may be reasons to adopt a more restricted concept of translation but we think it is unclear how this should be done. In general, we think that philosophers and cognitive scientists have been overly focused on the idea that mental representational systems are discrete, static systems defined by a distinct, unchanging format Evidence from cognitive neuroscience suggests that the brain has an extremely flexible ability to construct representational systems, that representational systems with different formats can overlap (auditory, visual, and haptic representational systems use shared spatial representations), that multiple formats can be encompassed in an integrated representational system (working memory is an integrated workspace which includes auditory, visual and other subcomponents), and so on (see Author 2020 for an extended discussion). Consequently, we doubt that it will be possible to draw a clear distinction between within-system and between-system content-relating processes. More specifically, it’s likely that content-relating and construction processes that operate within cognitive representational systems also play a role in constructing new representational systems and building relations across existing systems when this becomes functionally beneficial. These points have strong implications for understanding skill learning, which, we suggest, involves the construction of multiple interlinked domain-specific representational systems. In future work we’ll develop the idea that the processes we described here as translating between levels of abstraction play a key role in the formation of new representational systems for skill control.

9 Recent philosophical discussion has been preoccupied with a narrower 'interface problem' formulated by Butterfill and Sinigaglia (2014). The idea is that there is a special difficulty understanding how cognitive and motor representations can interact which stems from the supposed impossibility of translation between their formats. However, Christensen (2020) argues that the theoretical rationale for rejecting translation is unsound and that there is extensive empirical evidence that the brain translates across many representational formats, including motor and cognitive.

10 We won't engage in a comprehensive analysis of the DPM model here. Brozzo (2021) criticises the distinction between present- and future-directed intentions. Christensen (2020) gives an alternative account of motor representations and their interface with higher level intentions.

11 Although it should be noted that Shepherd's account includes a discussion of the role of domain norms in action evaluation which brings it closer to the problem-based conception.

12 This example reflects common advice to, and complaints about, inexperienced musicians. The following article provides a fairly typical illustration: https://www.premierguitar.com/articles/print/28953-last-call-space-is-music-too.

13 Our use of the term ‘system’ here may suggest that we have in mind a Fodorian module. However, we are adopting a different, non-Fodorian conception of modules and systems which is based in a biological framework. By ‘system’ we mean a network of structures and processes that show a degree of somewhat specific functional integration and perform one or more somewhat specific functional roles (in this case, action evaluation). A cognitive system in this sense can integrate with, and overlap with, other cognitive systems. The AES will be a ‘system of systems’ which encompasses multiple lower and higher level evaluative systems, for instance including both reward and efficiency evaluation systems.

14 In this respect our account can be contrasted with that of the predictive processing framework, which claims that behaviour is governed by a single dominant goal, namely predictive error minimization. Our account thus avoids the ‘dark room’ problem facing the predictive coding approach, which is that predictive error minimization can be achieved by placing oneself in a highly predictable environment (Sun & Firestone, 2020). We thank a referee for this point.

15 Drawing on Kath’s experience of teaching this exercise to beginner riders at skills clinics, many riders think they are almost about the graze the tyre when they are still approximately 20 cm away from reaching it. This was also true of Wayne. Kath communicated this to him verbally during the exercise, showed the gap with a hand gesture, and demonstrated the correct vs incorrect technique. In response, Wayne exaggerated his attempt at the task and was able to graze the rear tyre. Both riders were delighted!

16 Also called a ‘front wheel washout’. There are some differences in the way that ‘washout’ is conceptualised in the mountain bike community. The version of the concept Wayne was using treats it as any case in which the front wheel loses traction and slides out from underneath the rider, causing a crash.

17 This folk concept of flow should be differentiated from the concept used in psychology (Csikszentmihalyi 1990).
